# Analytical performance of a canine ELISA monocyte chemoattractant protein-1 assay for use in cats and evaluation of circulating levels in normal weight and obese cats

**DOI:** 10.1186/s13028-022-00640-3

**Published:** 2022-09-05

**Authors:** Kathrine Stenberg, Line Gensby, Signe Emilie Cremer, Michelle Møller Nielsen, Charlotte Reinhard Bjørnvad

**Affiliations:** 1 Department of Veterinary Clinical Sciences, Faculty of Health and Medical Sciences, University of Copenhagen, Dyrlægevej 16, 1870 Frederiksberg, Denmark; 2 Present Address: AniCura Vangede Animal Hospital, Plantevej 2, 2870 Dyssegård, Denmark; 3 Present Address: Coloplast, Holtedam 1-3, 3050 Humlebæk, Denmark

**Keywords:** Feline, Inflammation, MCP-1, Obesity, Sensitivity, Specificity, Validation

## Abstract

**Background:**

In human and murine obesity, adipose tissue dwelling macrophages and adipocytes produce monocyte chemoattractant protein-1 (MCP-1) leading to systemic low-grade inflammation. The aim of the study was to validate a canine MCP-1 ELISA assay for use in cats and to investigate whether a difference in MCP-1 concentrations could be detected between: a) cats having normal or elevated circulating serum amyloid A (SAA) levels and b) normal weight and obese cats. Serum obtained from 36 client-owned cats of various breed, age and sex with normal (n = 20) to elevated SAA (n = 16) was used for the validation of the canine MCP-1 ELISA assay. As no golden standard exists for measurement of inflammation, circulating MCP-1 concentrations were compared to SAA measurements, as an indicator of systemic inflammation. Analytical precision, dilution recovery and detection limit were calculated. A possible correlation between MCP-1 concentrations and obesity related measures (body fat percentage (BF%), insulin sensitivity and cytokine expression) were investigated in another population of 73 healthy, lean to obese, neutered domestic short-haired cats.

**Results:**

Intra- (2.7–4.1%) and inter-assay (2.2–3.6%) coefficient of variation and dilution recovery were acceptable, and the detection limit was 27.1 pg/mL. MCP-1 did not correlate with SAA, and there was no difference between the inflammatory (SAA > 20 mg/L) and non-inflammatory group, due to a marked overlap in MCP-1 concentrations. Circulating MCP-1 concentrations were unaffected by BF% (r^2^ = 2.7 × 10^–6^, P = 0.21) and other obesity-related markers.

**Conclusions:**

The present canine ELISA assay seems to be able to measure circulating feline MCP-1. However, further studies are needed to determine its possible use for detecting inflammation in relation to disease processes or obesity-related low-grade inflammation in cats.

## Background

Obesity is defined as an accumulation of excess body fat and is the most frequent nutrition related disease in cats [1], the prevalence of which varies between 34–45% depending on the study [2–4]. Based on studies in rodent models, it has been shown that, as obesity develops, the adipocyte expands to accommodate the accumulating triglycerides. Adipocyte growth causes compression of capillaries and the vasculature becomes insufficient to maintain normoxia in the adipose tissue. Combined with the growth restriction excerted by the extra cellular matrix, this leads to adipose tissue hypoxia, resulting in increased local fibrosis and adipocyte necrosis. A rise in adipocyte derived pro-inflammatory cytokines, such as tumor necrosis factor alpha (TNF-α), interleukin-6 (IL-6) and monocyte chemoattractant protein-1 (MCP-1), has been detected in humans and rodents [5, 6], which stimulates initiation of angiogenesis and increases blood supply counteracting hypoxia [7–9]. The adipocyte production of MCP-1 and adipokines such as leptin attracts monocytes to the adipose tissue in numbers which are proportional to the degree of obesity in humans and mice [10–12]. Once in the tissue, the monocytes differentiate into activated, cytokine producing macrophages [7, 10–16] increasing the systemic concentrations of inflammatory markers such as TNF-α and IL-6 [9, 17–20]. This leads to a chronic low-grade inflammatory state, which plays a central part in the pathogenesis of obesity related metabolic dysfunction and type 2 diabetes in humans and rodents [21, 22].

To investigate whether feline obesity leads to chronic low-grade inflammation, predisposing to metabolic dysfunction as in humans and rodents, previous studies have attempted to measure circulating IL-6 and TNF-α in cats. Some have detected an obesity related difference in cytokine levels [23], while others have not been able to confirm these findings [24–26]. One study found that the performance of cytokine ELISA assays used for feline studies were too poor for the estimation of MCP-1, IL-6 and TNF-α [27]. Although the search for an ideal marker of low-grade inflammation in cats has yielded variable results, serum amyloid A (SAA), an acute phase protein (APP), has been validated as a marker for acute systemic inflammation in cats [28–31]. It is a serum precursor of the main fibrillar component in reactive amyloid deposits, amyloid A, and is produced in the liver, thyroid and kidneys among other tissues [32]. Serum amyloid A has been shown to be a reliable marker of acute systemic inflammation in cats, increasing rapidly in the early stage of inflammation in several diseases and conditions such as acute pancreatitis, feline infectious peritonitis, cancer and feline lower urinary tract disease [28–31]. One study also found an increase in SAA in non-inflammatory conditions such as diabetes mellitus, chronic renal failure, hyperthyroidism and polycystic kidney disease in cats, possibly due to an underlying infection or vascular endothelium damage [31].

As a positive correlation between circulating MCP-1 and body mass index (BMI) and type 2 diabetes mellitus in humans and mice has been demonstrated [14, 33, 34], it is possible that MCP-1 could be used to quantify chronic low-grade inflammation in cats. If so, further investigations into the role of the association between circulating MCP-1 concentrations in relation to obesity related low-grade inflammation and insulin sensitivity in cats should be performed.

A feline MCP-1 assay was not available at the time of the study, but because of 89% homology between feline and canine MCP-1 protein [35], it was hypothesized that a commercial canine MCP-1 ELISA assay could prove usefull for measuring feline MCP-1 as well. The aim of this study was first to validate a commercial canine MCP-1 ELISA assay for measuring circulating feline MCP-1 by determining the analytical precision, dilution recovery and detection limit. Secondly, a possible correlation between MCP-1 concentrations and circulating levels of the acute phase protein SAA was evaluated in cats having normal or elevated circulating SAA levels. Thirdly, to test the hypothesis, that systemic MCP-1 concentration reflect obesity related low-grade inflammation, circulating MCP-1 levels were correlated to measured body fat percentages (BF%) in a group of lean to obese cats. Further, in the same cohort of lean to obese cats, circulating MCP-1 concentrations were correlated to MCP-1 mRNA levels in adipose tissue measured previously [36] as well as other inflammation associated genes i.e. plasminogen activator inhibitor-1 (PAI-1), tumor necrosis factor-α (TNF-α), interleukin-1β (IL-1β) and interleukin-6 (IL-6). Finally, the correlation between circulating MCP-1 levels and measures of insulin sensitivity (area under the curve for glucose (AUCg) and insulin (AUCi), homeostasis model assessment (HOMA) and quantitative insulin sensitivity check index (QUICKI)) were investigated.

## Materials

### Study populations

#### Population for studying analytical performance and correlation with circulating levels of SAA

The study was performed at the University Hospital for Companion Animals, University of Copenhagen, Denmark. The analytical performance of the ELISA assay and the correlation between measured MCP-1 and circulating SAA was evaluated using serum samples from 36 client-owned cats of various breed, age [median (range), 6 years (1–16 years)] and sex (n_female_ = 18, n_male_ = 18).

The samples were obtained for diagnostic purposes and analysed at the Veterinary Diagnostic Laboratory, Department of Veterinary Clinical Sciences, University of Copenhagen, Denmark. The blood samples were collected in the jugular or cephalic vein and centrifuged according to standard protocols. Following relevant diagnostic analyses, the remaining serum was stored in plastic vials at − 20 °C until inclusion in the present study, according to approval by the local ethical committee. The samples had previously been used as part of another study at our laboratory, validating a turbidimetric immunoassay for measurements of feline SAA as a marker of acute inflammation in cats [37]. Samples were only thawed when needed for analysis in order to limit freeze–thaw cycles. All included cats had a full physical examination and blood samples were collected for complete blood cell count and biochemistry analysis.

The 36 cats were divided into two groups, one with a SAA concentration below the established laboratory reference range [30] (normal SAA, n = 20, SAA < 5 mg/L) and one with elevated SAA concentrations indicating primary or secondary systemic inflammation [38] (elevated SAA, n = 16, SAA > 20 mg/L). Cats were excluded from the normal SAA group if they had received antibiotic or anti-inflammatory (non-steroidal anti-inflammatory drugs (NSAID) or prednisolone) treatment within the previous three weeks, because this could falsely decrease signs of systemic inflammation. The cats in the normal SAA group included cats with a history of vomiting (n = 1), asthma (n = 1), neurological symptoms (e.g. ataxia) (n = 2), epilepsy (n = 1), chronic kidney disease (CKD IRIS stage II-III based on circulating creatine levels [39], n = 4, range 171–259 µmol/L), hyperthyroidism (diagnosed by elevated circulating T4 and free T4 levels, n = 2), parasitic pulmonary infection (n = 1), heart murmur (n = 1) and healthy cats (n = 7). For the elevated SAA group, antibiotic/NSAID treatment was not an exclusion criteria. The elevated SAA group included cats with pyometra (n = 1), pyelonephritis (n = 1), pyothorax (n = 1), sepsis (n = 1), cancer (n = 1), pancreatitis and diabetes mellitus (diagnosed by glucosuria, hyperglycemia, hyperfructosanemia and positive SNAP fPLI, n = 1), abscess (n = 2), tumor of unknown type (n = 1), fracture (n = 1), vomiting (n = 1), foreign body in the intestinal tract (n = 1) and anemia (n = 1). The remaining three cats had increased SAA for unknown reasons. In this group, 10 cats received antibiotic treatment, five of which also received NSAID. Of note, multiple serum samples taken on separate days from three cats with elevated SAA concentrations were included in the analytical performance study to maximize the amount of serum available for the tests validation, resulting in 40 serum samples obtained from 36 cats.

#### Population for studying correlation between obesity, markers of tissue inflammation and insulin sensitivity

Serum samples from 73 healthy, adult (> 3 years of age), neutered, indoor confined, normal weight to obese domestic short-haired cats (Table 1), were analyzed for a possible correlation between circulating MCP-1 and fat mass, markers of tissue inflammation and insulin sensitivity.

**Table 1:** Characteristics of the study population (n = 73) divided into body fat percentage groups

Parameter	Normal weight (BF% < 35) (n = 26)	Overweight (35 ≤ BF% < 45) (n = 28)	Obese (BF% ≥ 45) (n = 19)	P value
Age, years	5.5 ± 2.1^a^	6.5 ± 2.7^b^	8.3 ± 2.7^c^	< 0.002
Sex, M / F, n^d^	12 / 14^a^	11 / 17^a^	11 / 8^a^	NS
Girth, cm	39 ± 4.1^a^	44 ± 3.7^b^	50 ± 3.9^c^	< 0.0001
Body weight, g	4584 ± 900^a^	5616 ± 1145^b^	7122 ± 1040^c^	< 0.0001
Fat mass, g	1373 ± 439^a^	2293 ± 553^b^	3520 ± 643^c^	< 0.0001
Lean mass, g	3211 ± 523^a^	3323 ± 636^a^	3602 ± 434^a^	< 0.06
BF%	29.3 ± 5.3^a^	40.6 ± 3.0^b^	49.2 ± 2.3^c^	< 0.0001

Characteristics of indoor confined, neutered, domestic short-haired cats (n = 73) divided into groups of normal weight (BF% < 35), overweight (35 ≤ BF% < 45) and obese (BF% ≥ 45) based on body fat percentage as measured by dual energy X-ray absorptiometry, also described in the previous study by Bjornvad et al. [40]. Values are given as mean ± SD for age, girth, body weight, fat mass, lean mass and BF%. Lean mass, fat mass, body weight and body fat percentage as measured by dual energy X-ray absorptiometry. Abbreviation: BF%, body fat percentage

^a, b, c^Values not sharing the same subscript are statistically significantly different

^d^Analysed using Chi-square test

The cats had been included in a previous study at the University Hospital for Companion Animals, University of Copenhagen, Denmark, and the sampling procedure has been described in detail elsewhere [40, 41]. In summary, at inclusion, owners signed a written informed consent, the cats had a physical examination, were weighed (Soehnle 8310, 0–20 kg, Murrhardt, Germany), had their girth measured and body condition score (BCS) estimated using a validated nine point scale [42]. The body composition of the cats was evaluated using dual energy X-ray absorptiometry (DEXA, Lunar DPX αlpha, GE Healthcare, Madison, WI, USA), estimating the individual BF%. Blood samples were obtained and serum was stored at -80 °C until further analysis. The cats were divided into three groups; normal weight (BF% < 35), overweight (35 ≤ BF% < 45) and obese (BF% ≥ 45) based on BF% as measured by DEXA [40]. A BF% of 35 was used as a cut off value between lean and overweight according to the previous reporting on the same cohort of indoor confined inactive cats [40], where cats with a BCS of 5/9, generally referring to normal weight, had a higher BF% (BF% range_BCS 5_, 23.6–38.4) than reported in previous studies and cats with BCS ≥ 6/9 had a BF% range of 28.3–55.4. Both sexes were equally distributed between the three groups normal weight (BF% < 35; n_male_ = 12, n_female_ = 14), overweight (35 ≤ BF% < 45; n_male_ = 11, n_female_ = 17) and obese (BF% ≥ 45; n_male_ = 11, n_female_ = 8).

### MCP-1 analysis

Monocyte chemoattractant protein-1 concentrations were analysed using the Canine ELISA CCL2/MCP-1 Quantikine® Immunoassay (R&D Systems, Minneapolis, Minnesota, USA) with an analytical range of 15.6–1000 pg/mL prior to any sample dilutions. The assay is a quantitative sandwich enzyme immunoassay technique employing a monoclonal canine specific antibody on a pre-coated microplate. The assay employs a canine MCP-1 conjugate and a canine positive control, which were supplied by the kit. A canine MCP-1 standard for the generation of a standard curve for the calculation of MCP-1 concentrations from the observed optical densities was also supplied by the kit. All reagents and procedures were prepared and performed according to manufacturer’s guidelines. All samples were run in duplicates and absorbance was estimated spectrophotometrically using SeptraMax® 340PC384 Microplate reader (Molecular Devices, Sunnyvale, CA, USA) at 450 nm and 650 nm wavelength. The results were read by the use of SoftMax®Pro software (Molecular Devices).

### Analytical performance

Monocyte chemoattractant protein-1 concentrations were measured in 40 serum samples from the 36 incuded cats to identify high, intermediate and low concentration intervals. Five serum samples from each MCP-1 concentration interval; high, intermediate and low were mixed to produce three pools with five serum samples in each pool. Intra-assay variation was calculated from five replicates, analyzed on the same kit. Inter-assay variation was estimated using five kits for the low and intermediate pools. Due to scarce amounts of serum, the high pool was only measured on four kits. The three pools were stored in separate vials at − 20 °C between each inter-assay analysis to reduce the number of freeze–thaw cycles. The dilution recovery of the assay was estimated using deming regression between the measured and expected MCP-1 concentrations. A 10% dilution series (ranging from 0 to 100%) of the high pool was produced using calibrator diluent provided with the assay. As a MCP-1 gold standard analysis for comparison of MCP-1 concentrations does not exist and as we were not able to obtain recombinant feline MCP-1 for spike/recovery analysis, a linearity check with high pool concentration of endogenous MCP-1 was performed. The detection limit of blank was established in 10 wells processed according to the assay procedure using assay diluent as blanks.

### SAA analysis

The commercially available human SAA turbidimetric immunoassay (SAA-TIA; LZ-SAA, Eiken Chemical Co., Tokyo, Japan) was used and the analyses were performed on an automated analyzer (ADVIA 1800 Chemistry System, Bayer, Newbury, UK) for the quantification of circulating feline SAA, as part of a previous study [37]. Serum amyloid A was used as a reference standard for the measurement of inflammation, as it has been found to be clinically valuable as a routine test to detect systemic inflammation in cats [31]. The assay has previously been validated for clinical use in cats [30].

### Adipose tissue gene expression

The quantification of the expression of several genes associated with inflammation (i.e. MCP-1, PAI-1, TNF-α, IL-1β and IL-6) was performed using quantitative polymerase chain reaction (qPCR) in another study on adipose tissue samples from the same feline study population [36]. Subcutaneous adipose tissue samples were obtained in full anaesthesia and snap frozen immediately after sampling for later processing. Extraction of mRNA from the adipose tissue samples was performed using the miRNeasy Mini Kit (Qiagen, Hilden, Germany) and mRNA concentration was measured spectrophotometrically and the measures were used for the calculation of the mRNA amount required for following cDNA synthesis. Feline specific primers and probes applied in the study were specifically developed for the study and developed by Microsynth (AG, Balgach, Switzerland) according to specifications for each gene of interest, and specific feline hydrolysis probes were developed for the Taqman assays. A Taqman protocol was applied for the genes; MCP-1 and PAI-1 and a manual SYBR green protocol was applied for IL-1β, IL-6 and TNF-α. Glyceraldehyde-3-phosphate dehydrogenase (GAPDH) was chosen as reference gene for both protocols. Samples, no-template controls, an internal feline adipose tissue control sample and a synthetic positive control sample (Microsynth) were included on all plates and run in triplicates. For both the Taqman and SYBR green protocol, qPCR was run at 50 °C for 2 min followed by 95 °C for 10 min and 50 cycles of denaturation at 95 °C for 15 s, followed by an annealing and extending step at 60 °C for 1 min. Product identities were confirmed by melt curve analysis in the SYBR green protocol. The gene expressions were quantified using the comparative Ct method [43].

### Insulin sensitivity measures

As described previously [41], an intravenous glucose tolerance test was performed on included cats and measures of insulin sensitivity were estimated. Insulin concentrations were measured and HOMA score, QUICKI and AUCg and AUCi were calculated. The cats were subjected to an intravenous glucose tolerance test (IVGTT) (glucose, 1 g/kg body weight IV) following 16 h of fasting. Blood samples were collected at specific times (0, 5, 10, 15, 30, 45, 60, 90 and 120 min.). Glucose concentrations were measured immediately after sampling and plasma was stored at -80 °C for later estimation of insulin concentrations determined using a heterologous immunoradiometric assay (INS-Irma; Biosource Europe S.A., Nivelles, Belgium) validated for use in cats [44]. As indices of insulin sensitivity, AUCg and AUCi were calculated, HOMA and QUICKI scores were calculated using the following formulas: HOMA score: (fasting serum glucose (Gt_0_,mmol/L) x fasting insulin concentration (It_0_, µU/L))/22.5 and QUICKI score: 1/((log (Gt_0_, mmol/L × 18.0182) + (log It_0_ µU/L)) [45, 46].

## Statistical analysis

Statistical analyses were performed using Microsoft Excel 2011 (Microsoft Corporation, Redmond, Washington, USA), Graph Pad Prism (Graph Pad Prism version 6.05, GraphPad Software, Inc., La Jolla, California, USA) and R statistical computing software (R, version 3.4.1; R Foundation for Statistical Computing, Vienna, Austria). For the analytical performance study, intra- and inter-assay coefficients of variation (CV) were calculated as: *CV* = *(standard deviation (SD)/mean)* × *100%*. The detection limit was estimated by applying the mean of the blank well measurements and the SD, using the following formula: *mean* + *3 SD*. For the dilution recovery analysis, normal distribution was achieved using log transformed data, and normality was tested using the Shapiro–Wilk normality test and QQ plots. Dilution recovery was calculated using deming regression analysis and Runs-test. Further analysis was performed using a Bland–Altman plot in which the difference between measured and expected MCP-1 concentrations was plotted against the mean concentration. The systematic bias was calculated as the mean of the differences ± SD.

A Mann–Whitney test was used for the investigation of difference in MCP-1 concentrations between the normal and elevated SAA group. After log transformation, a linear regression analysis with MCP-1 as the outcome variable was performed for the association between SAA and MCP-1 concentrations. Residual diagnostics showed that the residuals were normally distributed after log transformation and no overtly influential observation was noted. A Student's unpaired t-test was used for the investigation of difference in age, weight, girth, fat mass, lean mass and BF% between males and females (n_cats_ = 36). A Chi-square test was performed for the analysis of difference in BCS between males and females.

A one-way ANOVA using Tukey's multiple comparisons test was performed for the investigation of difference in age, weight, girth, fat mass, lean mass and BF% between BF% groups following testing for normality. A Chi-square test was performed for the analysis of difference in BCS between BF% groups. Furthermore, a multiple linear regression with Likelihood ratio test was performed, including BF% group, age, BCS and gender as explanatory variables. For the investigation of the correlation between circulating MCP-1 concentrations and BF%, the tissue specific mRNA levels of several inflammatory genes and the measures of insulin sensitivity, a Spearman correlation test was performed. The level of statistical significance in all tests was set at 0.05. Results are presented as mean ± SD unless otherwise stated.

## Results

### Analytical performance

**Fig. 1:**
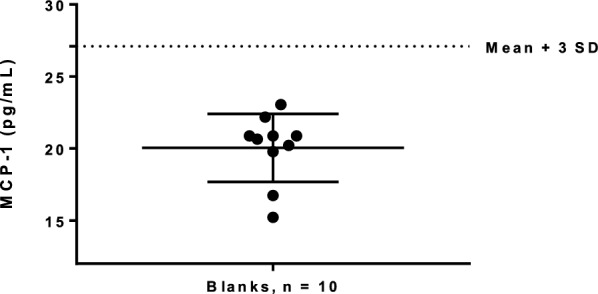
Monocyte chemoattractant protein-1 (MCP-1) concentrations (pg/mL) measured in blank wells (n = 10). Mean ± SD is indicated by the full black lines. Detection limit is indicated by the horizontal dotted line

**Fig. 2:**
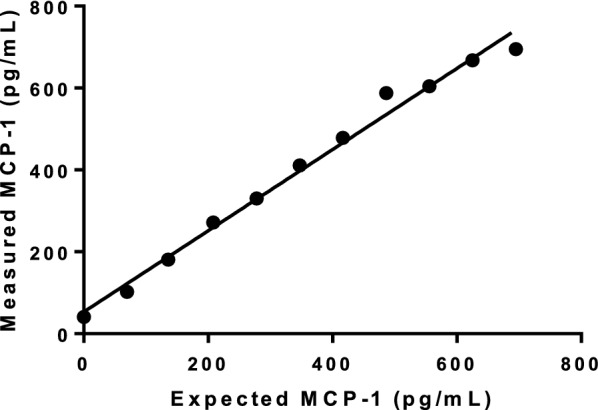
The dilution recovery of a canine monocyte chemoattractant protein-1 (MCP-1) ELISA assay used for measuring feline circulating concentrations depicted as a deming regression line. The measured MCP-1 concentration (pg/mL) is plotted on the Y-axis against the expected MCP-1 concentration on the X-axis (n = 11, Y = 0.997 X + 51.23; 95% CI_Y-intercept_, 18.0–84.4; 95% CI_slope_; 0.92–1.08). Abbreviations: CI, confidence interval

**Fig. 3:**
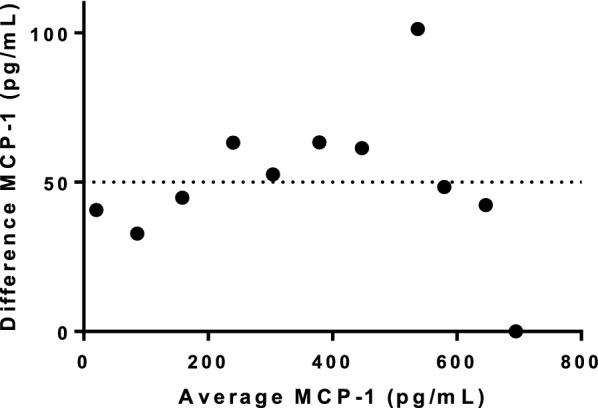
Bland–Altman plot of the difference in measured monocyte chemoattractant protein-1 (MCP-1) concentrations (pg/mL) in a high pool 10% dilution series compared to the expected MCP-1 concentration (n = 11). The difference between the measured and the expected concentrations are plotted on the Y-axis and the mean values are plotted on the X-axis. The mean of the bias is indicated by the dotted horizontal line

Prior to producing three pools (high, intermediate and low MCP-1 concentrations) using five serum samples in each pool, the MCP-1 concentrations of the samples used for the development of the three pools were analyzed (range _high pool_, 350–831.6 pg/mL; range _intermedium pool_, 167.5–241.1 pg/mL; range _low pool_, 55.2–78.0 pg/mL). Repeated pool measurements revealed observed MCP-1 intra-assay CVs of 2.7–4.1% and inter-assay CVs of 2.2–3.6% (Table 2). The pools were measured in replicates of five for both intra-and inter-assay variations, however due to a scarcity of serum, the high pool was only measured four times for the inter-assay variation. The detection limit of blank for the MCP-1 assay was estimated to be 27.1 pg/mL (Fig. 1). Dilution recovery was calculated between the expected and measured MCP-1 concentrations of the dilution series (Fig. 2). The slope was not significantly different from one (slope, 95% CI; 0.997, 0.92–1.08). A Runs-test analysis showed that the results deviated from the linear model (P = 0.02) and the data was therefore analyzed using a Bland–Altman plot which yielded a systematic bias of 50.0 pg/mL ± 24.7 pg/mL (Fig. 3).

**Table 2:** Observed intra- and inter-assay variations of feline monocyte chemoattractant protein-1 concentrations (MCP-1)

	No. runs	Mean (pg/mL)	SD (pg/mL)	CV (%)
Intra-assay				
Low Pool	5	95.1	3.3	3.5
Medium Pool	5	257.4	6.9	2.7
High Pool	5	719.1	29.6	4.1
Inter-assay				
Low Pool	5	82.2	2.7	3.4
Medium Pool	5	232.1	8.2	3.6
High Pool	4	691.7	15.9	2.2

Observed intra- and inter-assay variations of feline monocyte chemoattractant protein-1 concentrations (MCP-1) within and between kits

*SD* Standard diviation, *CV* Coefficient of variation

As the investigation of the assay's analytical performance yielded acceptable precision, accuracy and detection limit, an analysis of MCP-1 concentrations in cats with normal and elevated SAA was performed.

### Analysis of correlation between MCP-1 concentrations and SAA groups and measurements

**Fig. 4:**
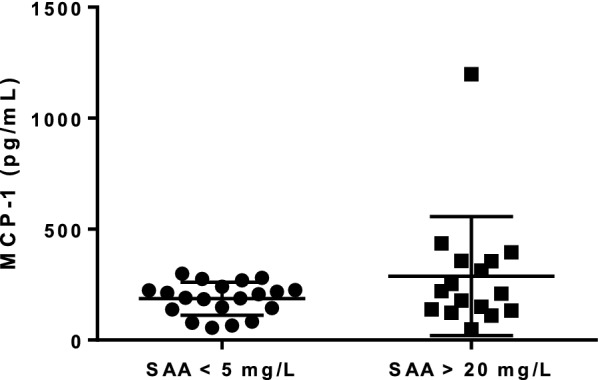
Distribution of monocyte chemoattractant protein-1 (MCP-1) serum concentrations (pg/mL) in cats without systemic inflammation (n = 20, serum amyloid A (SAA) < 5 mg/L) and cats with systemic inflammation (n = 16, SAA > 20 mg/L). Mean ± SD are depicted as the horizontal lines, P = 0.35

No difference in MCP-1 concentrations was observed between the normal SAA group (186.5 pg/mL ± 74.0 pg/mL) and the elevated SAA group (288.7 pg/mL ± 268 pg/mL, Fig. 4). The association between circulating MCP-1 and SAA concentrations was not statistically significant (r^2^ = 0.03, P = 0.3).

### Analysis of correlation between MCP-1 concentrations and BF%

Characteristics of the indoor confined, neutered, domestic short-haired cats (n = 73) divided into groups of normal weight (BF% < 35), overweight (35 ≤ BF% < 45) and obese (BF% ≥ 45) based on body fat percentage as measured by dual energy X-ray absorptiometry, also described in the previous study by Bjornvad et al*.* [40], are presented in Table 1.

**Fig. 5:**
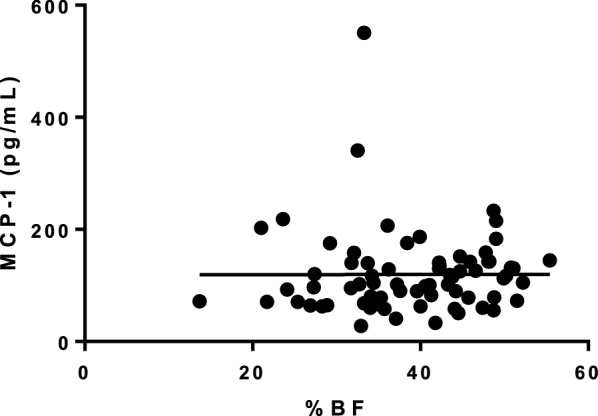
Correlation analysis of the serum monocyte chemoattractant protein-1 (MCP-1, pg/mL) and body fat percentage (BF%) as measured by dual energy X-ray absorptiometry. r^2^ = 2.7 × 10^–6^, P = 0.21

Circulating MCP-1 concentrations did not correlate with BF% (r^2^ = 2.7 × 10^–6^, P = 0.21) (Fig. 5). MCP-1 concentrations were not influenced by BF%, age, BCS or sex (P = 0.65, P = 0.68, P = 0.21 and P = 0.14, respectively) in the linear multiple regression analyses.

### Correlation between circulating MCP-1 concentrations and adipose tissue gene expression of inflammatory markers

Circulating MCP-1 concentrations correlated weakly with MCP-1 and PAI-1 mRNA gene expression in the adipose tissue (MCP-1; r^2^ = 0.04, P = 0.008; PAI-1; r^2^ = 0.09, P = 0.001, Table 3). The circulating MCP-1 concentrations did not correlate with the remaining investigated genes associated with inflammation i.e. TNF-α, IL-1β and IL-6 (Table 3).

**Table 3:** Correlation between circulating monocyte chemoattractant protein-1 concentrations and adipose tissue gene expression of inflammatory markers analyzed using the Spearman correlation test

Parameter	r^2^	P value
MCP-1	0.04	0.008
PAI-1	0.09	0.001
TNF-α	0.01	NS
IL-1β	1.5 × 10^–5^	NS
IL-6	0.006	NS

*MCP-1* Monocyte chemoattractant protein-1, *PAI-1* Plasminogen activator inhibitor-1, *TNF-α* Tumor necrosis factor-α, *IL-1β* Interleukin-1β, *IL-6* Interleukin-6

### Correlation between circulating MCP-1 concentrations and insulin sensitivity measures

The results for HOMA and QUICKI between BF% groups and sexes have previously been published [41]. There was no correlation between circulating MCP-1 concentrations and the measures of insulin sensitivity; QUICKI, HOMA and AUC_i_. A very weak correlation was detected between MCP-1 and AUC_g_ (r^2^ = 0.009, P = 0.04).

## Discussion

The commercial canine MCP-1 ELISA assay showed to have good analytical performance in measuring feline circulating MCP-1. Circulating MCP-1 concentrations correlated weakly with measured fat tissue mRNA levels of both MCP-1 and PAI-1, but not with measures of more acute inflammation such as circulating SAA, and tissue mRNA expression of different cytokines. Monocyte chemoattractant protein-1 may be a marker of low-grade inflammation, but in the current cohort of lean to obese cats there was no correlation with obesity measured by BF%. A weak correlation was identified between circulating MCP-1 concentration and AUC_g_ while other measures of insulin sensitivity were unrelated.

### Analytical performance

The current study shows, that the canine MCP-1 ELISA assay can be used for measuring MCP-1 reliably in feline serum. The kit performed with high precision and acceptable dilution recovery. The intra- and inter-assay CVs were lower compared to a previous study, using the same canine assay [47], and within the recommended acceptable limits of 5% for automatized and 10% for manual kits, respectively [48]. The difference in measured compared to expected MCP-1 concentrations was above the analytical variance, and a constant systematic bias was identified, slightly overestimating the MCP-1 concentration. The detection limit of blank (for feline MCP-1) was higher than the canine detection limit reported by the manufacturer (10.8 pg/mL) [49]. The detection limit in the current study was calculated differently than by the manufacturer (3SD + mean of replicate assay diluent vs. 2SD + mean of replicate standard zero, respectively), which may explain the differences observed. In a previous feline study using the present assay, a validation or a detection limit was unfortunately not described [47]. As no samples in the current study were below the detection limit, it was assessed to be acceptable. Also, with a 89% homology between canine and feline MCP-1 [35], it is highly unlikely that the assay does not measure feline MCP-1. To investigate this further, a Western blot analysis could be performed using feline serum and the assay specific canine antibody. Unfortunately, the antibodies are considered proprietary, and could not be obtained for further testing. Another limitation to the study is that a comparision with a gold standard MCP-1 analysis was not possible as a gold standard currently does not exist for feline MCP-1. As we were not able to obtain recombinant feline MCP-1 for spike/recovery analysis, we performed linearity check with high pool concentration of endogenous MCP-1, and could conclude that the assay performs with acceptable accuracy. We cannot, however, know whether the assay has systematic bias compared to the ‘true’ concentration measured by a gold standard (i.e. over- or underestimates), but in this study, the conditions of the analysis were standardized for all analyzed groups and thus comparable.

Previous attempts to measure subtle changes in feline inflammatory cytokines as markers of potential obesity-associated low-grade inflammation have shown variable results [24, 25, 50]. One study found that available assays performed analytically too poorly [27]. Validated inflammatory markers are therefore highly warranted for investigating a possible feline obesity related chronic low-grade inflammation.

### MCP-1 concentrations in cats with normal or elevated SAA concentrations

There was no difference in MCP-1 concentrations between groups with normal and elevated SAA levels and no correlation between MCP-1 and SAA concentrations. This indicates that an increase in SAA concentration is not necessarily accompanied by an increase in MCP-1 in the cat and that SAA and MCP-1 are likely regulated by different mechanisms. Serum amyloid A is produced in the liver as part of a systemic acute phase response, whereas MCP-1 is secreted by endothelial cells, macrophages and adipocytes in response to local pro-inflammatory stimuli [51, 52]. The markers may therefore not be systemically present and measureable at the same point in time. Three high MCP-1 measurements (range; 831.59–1305.23 pg/mL) used for the analytical performance study were from consecutive samples obtained on separate days from the same cat diagnosed with pyothorax. In chronic pulmonary diseases in humans, increased locally expressed SAA can stimulate monocyte differentiation and lead to the production of pro-inflammatory cytokines [53]. Human studies have described an SAA stimulated increase in MCP-1 production in the smooth muscle of the vasculature and in sites of atherosclerosis contributing to a pro-inflammatory state in coronary artery disease [54, 55]. Furthermore, in human and murine studies, MCP-1 plays a central role in the exudative pleural fluid formation in pulmonary cancer and infections, and MCP-1 levels in the effusion correlate positively with effusion volume [56, 57]. Although atherosclerosis is not common in cats, it is possible that feline inflammatory respiratory diseases, such as pyothorax, could elicit a higher MCP-1 response than the other conditions represented in the elevated SAA group.

The chosen cut-off value for SAA could be an explanation for the lack of MCP-1 difference between the normal and elevated SAA groups, i.e. the chosen cut-off value was unable to clearly distinguish between cats with normal or elevated MCP-1 concentrations. The SAA cut-off was based on a previous study [30], in which median SAA concentrations for healthy cats were 0.4 mg/L (range; 0.0–3.9 mg/L) and 46.6 mg/L (range; 1.2–150.6 mg/L) for cats with systemic inflammation. The overlapping MCP-1 concentrations could also be due to cats in the elevated SAA group not having sufficiently severe inflammatory conditions. Further, cats treated with antibiotics and/or anti-inflammatory drugs were included in the elevated SAA group. This could have resulted in decreased inflammation and lower MCP-1 concentrations, as a study on obese rats found a decrease in MCP-1 concentration after treatment with cyclooxygenase 2 inhibitors [58]. Due to a scarcity of healthy cats, cats with a variety of conditions were included in the normal SAA group based on SAA levels within the reference interval. Some of the cats included had conditions that have been reported to increase SAA, such as hyperthyroidism and CKD.

Previously, in the obesity cat cohort the possible presence of macrophage infiltration in the feline adipose tissue was investigated by histological examination of formalin fixed subcutaneous adipose tissue samples [59]. Unfortunately, the integrity of the adipose tissue biopsies did not permit the quantification of macrophages or adipocytes. Two feline studies have investigated macrophage infiltration in the adipose tissue of obese cats [60, 61]. One study in kennel cats found no correlation between number of adipose tissue macrophages and degree of obesity or adipose tissue depot location [60]. Similiarly, in the other study, the number of adipose tissue-macrophages were not influenced by obesity but there was an increased activation of the adipose tissue residing macrophages in the obese cats [61]. These studies indicate that macrophage infiltration in the adipose tissue may not be a central feature of obesity in cats.

### MCP-1 concentrations in relation to BF% and insulin sensitivity

In the current study, MCP-1 levels were not correlated with BF%. This could indicate that feline obesity does either not elicit low-grade inflammation as observed in humans and rodents or that a possible low-grade inflammation is not driven by MCP-1 production. Studies in overweight and obese dogs have found a decrease in circulating MCP-1 concentrations during and after weight loss, although an overlap in concentrations between groups was observed [62–64]. Another canine study found no difference in circulating MCP-1 concentrations between lean and overweight dogs, only between lean and obese dogs [65]. The MCP-1 concentrations seem to be comparable in cats and dogs, although in the current study, the range was larger than observed in the canine studies. The current study shows that the canine assay can be used for measuring feline MCP-1 levels within the range 27.5–550.7 pg/mL. Similar to previous studies in other species [33, 62–65], there was a considerable overlap between groups and it is possible that feline obesity does not elicit sufficiently pronounced inflammation to result in an increase in circulating MCP-1 concentrations. If there truly is a lack of an obesity associated increase in circulating MCP-1, this might indicate that significant macrophage infiltration of the adipose tissue does not occur in feline obesity. This hypothesis is supported by the lack of correlation between adipose tissue MCP-1 gene expression and BF% and the weak correlation between circulating MCP-1 and adipose tissue MCP-1 gene expression.

The lack of obesity associated increase in circulating MCP-1 levels in the current cohort could be due to only a subtle obesity-associated difference in MCP-1 concentrations. Furthermore, the mean BF% of the normal weight cats was close to the lower limit for overweight cats in two previous publications [66, 67]. It is therefore possible that the body fat content of the normal weight cats was too high to distinguish their MCP-1 levels. However, the BF% and fat mass were still significantly higher in the overweight and obese BF% groups compared with the normal weight cats, and because a significant direct correlation between MCP-1 and fat mass has been reported in human and rodent studies [10, 68–70], a correlation was therefore expected in the current study.

Although obesity affected HOMA and QUICKI in the current cohort [41], no correlation was found between circulating MCP-1, and AUC_g_, HOMA and QUICKI, as measures of insulin sensitivity and only a weak correlation between circulating levels of MCP-1 and AUC_i_ was found. In human [71, 72] and murine studies [14, 73, 74] MCP-1 has been correlated with insulin resistance in some studies, while other studies found no correlation between obesity related inflammatory cytokines, including MCP-1, and the patient’s insulin sensitivity [75, 76].

A limitation of the present study was an uneven age distribution within the three BF% groups; the obese cats were older than the normal and overweight cats. However, because MCP-1 concentrations were not correlated with age in the current study and age has not been reported as a confounding factor in human or rodent studies, it seems unlikely that the slightly older age of obese cats affected the results. With respect to presence of systemic inflammation, SAA has shown to have varying correlation to other APPs, due to the APPs being secreted by different organs. A strong correlation has been found between SAA and another feline APP, α1-acid glycoprotein (AGP) [31]. SAA is produced in the liver, thyroid and kidneys [32] and AGP is secreted by the liver as well as by leukocytes in the chronic phase [77], which may explain a difference in the behavior of APPs in the chronic and acute inflammatory state. It is possible that another APP could reflect feline chronic low-grade inflammation better, but in general, markers of acute and chronic inflammation in cats have so far only been sparsely evaluated and the search for markers of feline chronic inflammation should continue.

## Conclusion

The current study shows that the canine MCP-1 ELISA assay may possibly be applicable for the measurement of MCP-1 in feline serum. However, a possible association between obesity and MCP-1 could not be detected in this cohort of lean to obese cats using the chosen commercial canine ELISA assay and the lack of correlation with SAA could suggest that MCP-1 is not an ideal marker of general acute circulating inflammation in cats in a clinical setting. These results do not entirely exclude the possibility of a systemic low-grade inflammation, but they do indicate that a possible obesity associated systemic low-grade inflammation in cats is probably not driven by MCP-1 as the main instigator.

Further investigation of other biomarkers in the continued search for measurable markers for low-grade inflammation in cats is warranted, as are investigations into which inflammatory conditions affect MCP-1 concentrations, for it to be applied as a potential inflammatory marker in the clinical setting and be of diagnostic relevance for the veterinary clinician.

## Data Availability

The datasets used and/or analysed during the current study are available from the corresponding author on reasonable request.

## Abbreviations

BCS: Body condition score
BF%: Body fat percentage
CV: Coefficients of variation
DSH: Domestic shorthaired cats
F: Female
M: Male
MCP-1: Monocyte chemoattractant protein-1
NS: Not significant
SAA: Serum amyloid A
TNF-α: Tumor necrosis factor-α
